# Supplementary material for: Identification of photoperiod-induced specific miRNAs in the adrenal glands of Sunite sheep (Ovis aries)
Source: Front Vet Sci. 2022 Jul 22;9:888207. doi: 10.3389/fvets.2022.888207 (PMC9354845; doi:10.3389/fvets.2022.888207)
Supplement: Supplementary file 1 [file Data_Sheet_1.ZIP › Supplementary file/miRDeep2 file.zip!/miRDeep2 file/Know_Structure/LP42A2/oar-mir-376c.pdf]

miRBase precursor : oar-mir-376c
Total read count : 772
oar-miR-376c-5p read count : 324
oar-miR-376c-3p read count : 447
remaining reads : 1

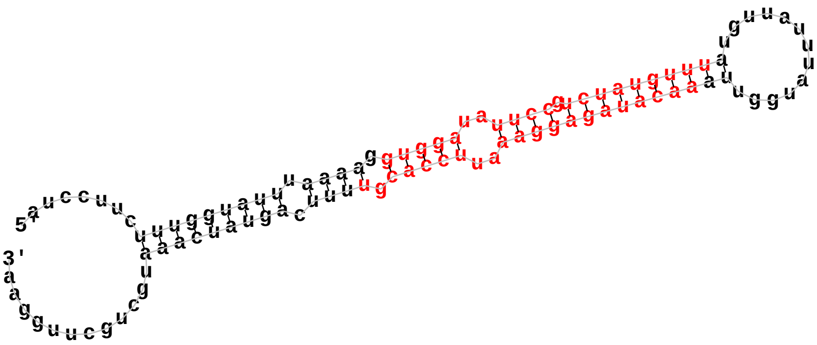

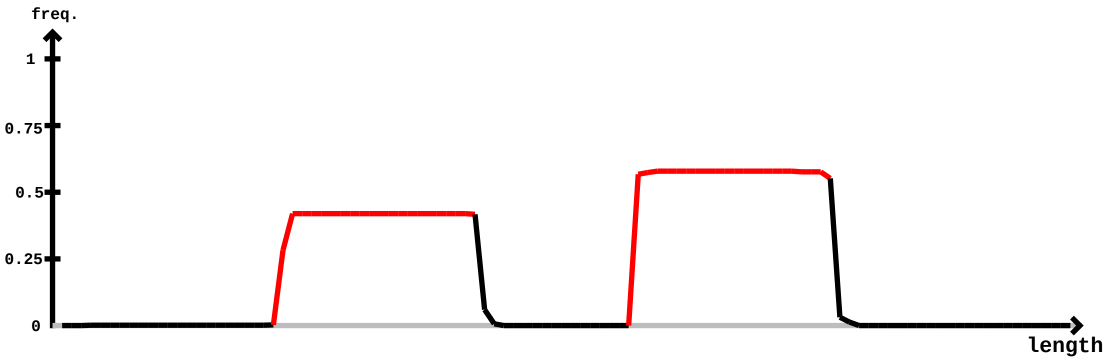

oar-miR-376c-3p

oar-miR-376c-5p

```
5'- auccuucuuugguauuuaaaagguggauauuccgucuauguuuauguuauuuaugguuaaacauagaggaaauuccacguuuucaguaucaaaugcugcuuggaa  -3'    exp
    .......(((((((((.((((.(((((..((((.((((((((((.............))))))))))))))..))))).)))).))))))))............    reads    mm    sample
    ..ccuucuuugguauuuaaaag...................................................................................    1        0     seq
    .....................gguggauauuccgucuauguuu..............................................................    1        0     seq
    ......................guggauauuccgucuauguu...............................................................    2        0     seq
    ......................guggauauuccgucuauguuu..............................................................    186      0     seq
    ......................guggauauuccgucuauguuua.............................................................    27       0     seq
    ......................guggauauuccgucuauguuuau............................................................    2        0     seq
    .......................uggauauuccgucuauguuu..............................................................    89       0     seq
    .......................uggauauuccgucuauguuua.............................................................    14       0     seq
    .......................uggauauuccgucuauguuuau............................................................    3        0     seq
    ...........................................................aacauagaggaaauucA.............................    1        1     seq
    ...........................................................aacauagaggaaauucc.............................    1        0     seq
    ...........................................................aacauagaggaaauuccacg..........................    18       0     seq
    ...........................................................aacauagaggaaauuccacgu.........................    402      0     seq
    ...........................................................aacauagaggaaauuccacguu........................    14       0     seq
    ...........................................................aacauagaggaaauuccacguuu.......................    2        0     seq
    ............................................................acauagaggaaauuccacg..........................    1        0     seq
    ............................................................acauagaggaaauuccacguuu.......................    4        0     seq
    .............................................................cauagaggaaauuccacguuu.......................    4        0     seq
```
